# Research across the disciplines: a road map for quality criteria in empirical ethics research

**DOI:** 10.1186/1472-6939-15-17

**Published:** 2014-03-01

**Authors:** Marcel Mertz, Julia Inthorn, Günter Renz, Lillian Geza Rothenberger, Sabine Salloch, Jan Schildmann, Sabine Wöhlke, Silke Schicktanz

**Affiliations:** 1 Institute for History of Medicine and Medical Ethics, Research Unit Ethics, University of Cologne, Herderstr. 54, D-50931 Cologne, Germany; 2 Institute for Ethics, History and Philosophy of Medicine, Hannover Medical School, Carl-Neuberg-Str. 1, D-30625 Hannover, Germany; 3 Department of Medical Ethics and History of Medicine, University Medical Center Göttingen, Humboldtallee 36, D-37073 Göttingen, Germany; 4 Protestant Academy Bad Boll, Bad Boll, Akademieweg 11, D-73087 Bad Boll, Germany; 5 Formerly at: Institute of Ethics and History in Medicine, Centre for Medicine, Society and Prevention, University of Tübingen, Gartenstr 47, D-72074 Tübingen, Germany; 6 Institute for Medical Ethics and History of Medicine, NRW Junior Research Group “Medical Ethics at the End of Life: Norm and Empiricism”, Ruhr University Bochum, Malakowturm, Markstr 258a, D-44799 Bochum, Germany

**Keywords:** Empirical ethics, Evidence-based ethics, Empirical methodology, Applied bioethics, Interdisciplinarity, Methodology, Quality criteria

## Abstract

**Background:**

Research in the field of *Empirical Ethics* (EE) uses a broad variety of empirical methodologies, such as surveys, interviews and observation, developed in disciplines such as sociology, anthropology, and psychology. Whereas these empirical disciplines see themselves as purely descriptive, EE also aims at normative reflection. Currently there is literature about the quality of empirical research in ethics, but little or no reflection on *specific* methodological aspects that must be considered when conducting *interdisciplinary* empirical ethics. Furthermore, poor methodology in an EE study results in misleading ethical analyses, evaluations or recommendations. This not only deprives the study of scientific and social value, but also risks ethical misjudgement.

**Discussion:**

While empirical and normative-ethical research projects have quality criteria in their own right, we focus on the specific quality criteria for EE research. We develop a tentative list of quality criteria – a “road map” – tailored to interdisciplinary research in EE, to guide assessments of research quality. These quality criteria fall into the categories of *primary research question, theoretical framework and methods*, *relevance*, *interdisciplinary research practice* and *research ethics and scientific ethos.*

**Summary:**

EE research is an important and innovative development in bioethics. However, a lack of standards has led to concerns about and even rejection of EE by various scholars. Our suggested orientation list of criteria, presented in the form of reflective questions, cannot be considered definitive, but serves as a tool to provoke systematic reflection during the planning and composition of an EE research study. These criteria need to be tested in different EE research settings and further refined.

## Background^a^

### Empirical ethics

For roughly two decades there have been debates in bioethics about the question of how to address the challenge of best practice in interdisciplinary methodology. Empirical research in bioethics, principally using the methods of social sciences [1,2]^b^, has considerably increased during this period (e.g. [3]).

Generally speaking, this debate comes under the label of what is known as “empirical ethics” (abbreviated “EE”, e.g. [4-8]); some authors prefer to talk about “empirically informed ethics” or sometimes “evidence-based ethics” (e.g. [9-13]). This field calls for more empirical research, mainly from sociology, psychology or anthropology, and/or more consideration of empirical research results in normative bioethics^c^. Whereas empirical disciplines aim to be purely descriptive, however, EE has a strong normative objective: empirical research in EE is not an end in itself, but a required step towards a normative conclusion or statement with regard to empirical analysis, leading to a *combination* of empirical research with ethical analysis and argument.

### Research problem

The widespread use of EE highlights the importance question above: what is the best practice for applying empirical methodologies in such an interdisciplinary setting? This interdisciplinary challenge is still not solved, and proponents of EE can self-critically assume that the quality of EE studies is often unsatisfactory. This problem can be tackled by two strategies: either focusing only on one particular methodology, or trying to establish standards for ‘good’ EE research. The advantage of the latter is obvious: methodologies are highly dependent on theoretical assumptions, and there is no such thing as one true theory, neither in empirical research nor in ethics. It therefore seems most appropriate to focus on best practice instead of perfecting one particular methodology in EE.

The lack of standards for assessing and safeguarding quality is not only a problem for scientific quality *per se*, but also an *ethical* problem: poor methodology in EE may give rise to misleading ethical analyses, evaluations or recommendations^c^, not only depriving the study of scientific and social value, but also risking ethical misjudgement. Improving the quality of EE is therefore an ethical necessity in itself.

### Aims & premises

This article aims to provide a “road map” (see below) to assist researchers in conducting EE research, and also to initiate a more focused debate within bioethics about how to improve the quality of EE research. Our contribution should be understood primarily as a *heuristic* approach. As the discussion on quality criteria for EE research is rather new and touches on a number of complex topics within interdisciplinary research we would see our article as a first and provisional suggestion in this respect. We will discuss four domains of quality criteria and provide a tentative list of questions to be considered by researchers when engaging in EE research. Each formal quality criterion will therefore be guided by practical questions which illustrate its reflective and methodological purpose.

In this paper we will focus mainly on providing and discussing the abstract criteria, but will refrain from citing detailed examples for each criterion because of length limitations. While different application fields for quality criteria can be imagined (such as journal peer review, assessment of research proposals, or the planning of individual research projects), it should be noted that the criteria we present are only designed for *guiding EE research* (and, partially and indirectly, for reporting on it, since the reported study is what peer reviewers and readers of scientific literature ultimately see).

We start from the premise – supported by our own research, and corroborated by several authors in the debate e.g. [6,8,14-18] – that empirical research is vital for the vast majority of normative ethical research. Here we focus on “applied ethics”, that is, research concerning analyses, evaluations and recommendations in ethically sensitive fields such as medicine and clinical research, genetics and neuroscience, and also economics and the media.

As far as empirical research is concerned, we limit our claims here to socio-empirical research, i.e. studies based on methodologies from the *social sciences*. As to the normative-ethical aspect of EE research, we primarily refer to normative-ethical research based on *philosophical* methods. While theological methods are also important and valuable, we did not assess them in the context of this work.

### Approach

#### Definition of empirical ethics research

As a descriptive definition of EE could not claim to define “empirical ethics” for all instances in which this term is used for this, (see e.g. [11,15,19]) we will confine ourselves to a *stipulative definition* covering the various ways of conducting EE research (e.g. [6,14,17,20,21]).

EE research, as we understand it, is normatively oriented bioethical or medical ethical research that directly *integrates* empirical research^e^. Key elements of this kind of study are therefore that it encompasses (i) empirical research as well as (ii) normative argument or analysis, and (iii) attempts to integrate them in such a way that knowledge is produced which would not have been possible without combining them. Concerning (iii), we proceed on the assumption that descriptive and normative statements can and should be analytically distinguished from each other in order to evaluate their validity [22-24]. Some proponents of EE, e.g. those taking a phenomenological or hermeneutical approach (e.g. [7,25-27]), would assume that descriptive and normative statements are inevitably inseparable and indistinguishable. However, in the context of the current article, we exclude from our analysis approaches to EE research which are mainly hermeneutically or historically oriented. We believe that they can fruitfully contribute to EE research, but these approaches are in need of specific quality criteria that go beyond the scope of this paper. Nonetheless, the development of quality criteria or best practice standards might also be relevant for these approaches.

The above-mentioned integration of empirical research and a normative-ethical argument makes interdisciplinary work inevitable. It implies collaboration between researchers trained in different fields and methodologies. While it is theoretically possible for interdisciplinary research to be carried out by a single researcher skilled in more than one academic field, most EE research will benefit from interdisciplinary research teams (e.g. [8]). This is because the skills needed for applying both sound empirical research methods and thorough normative analysis and argument are seldom possessed by a single researcher.

Working in teams also offers the opportunity to overcome methodological biases, penchants for particular research approaches and intellectual myopia in terms of background assumptions. For example, in qualitative research (e.g. interviews, observations), intersubjective exchange during the interpretation process is a necessary precondition for enhancing the validity of the results. It also seems unlikely, on the basis of the criteria we are about to present, that such interdisciplinary (team-) work can be done (fully) independent of other team members, based on a strict division of labour between empirical researchers and ethicists. For all these reasons, in our further analysis we proceed on the assumption that EE research should best be carried out in an interdisciplinary research team.

#### “Road map” analogy

We propose to use the analogy of a “road map” in order to structure the different criteria in our paper, applying the metaphor of moving through a (not yet familiar) landscape for the conduct of EE research. According to this metaphor, the following criteria can be understood as “landmarks”, indicating what paths to take, how fast to go, and where to expect a rocky road or a dead end.

#### Mapping landmarks of quality and drafting a road map

**Figure 1:**
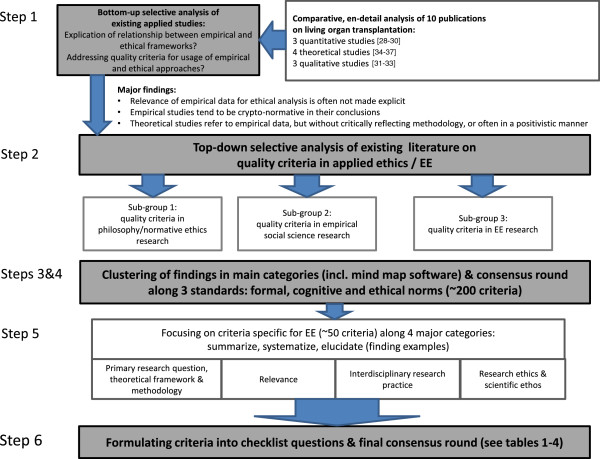
Search and analysis strategy.

To survey specific “landmarks” of quality in EE research, and to draft a corresponding “road map”, our procedure consists of the following main steps (Figure 1*Search and analysis strategy*, provides a graphical overview of the search and analysis strategy the working groups used during the project):

(i) to analyse selected empirical quantitative and qualitative studies as well as theoretical ethics studies about living organ donation (“bottom-up-strategy”) regarding their use of empirical data and ethical concepts, and if they reflected upon that relationship;

(ii) to study, present and critically discuss already established quality criteria for each of the following three branches of relevant criteria, *viz.* a) empirical/social science research, b) philosophical/normative-ethical research, and c) EE research (“top-down-strategy”);

(iii) to consider, present and critically discuss research ethics criteria for each of the three branches, in the light of our experience in EE research and knowledge of the EE debate;

(iv) to develop a consensus among the authors;

(v) to refine the different branches and reduce complexity for publication; and

(vi) to draft a tentative checklist of questions which operationalises criteria pertinent to EE research.

Our search and analysis strategy included first (i) a bottom-up analysis of 10 publications, dealing explicitly and/or implicitly with ethical and empirical issues of living organ donation. This field was used as a focused case study to allow a comparison of quantitative (n = 3 [28-30]) as well as qualitative (n = 3 [31-33]) empirical studies and theoretical ethics publications (n = 4 [34-37]).

This bottom-up detailed analysis revealed that firstly, the relevance of empirical data for ethical analysis is often not made explicit, secondly, empirical studies tend to be crypto-normative in their conclusions (“crypto-normative” for us implies that implicit evaluations and ethical conclusions are made, but the evaluative step is not explicated), and thirdly, theoretical studies often refer to empirical data, but rarely critically reflect the empirical methodology, or often tend to apply empirical data in a positivistic manner.

For step (ii), we applied a more top-down strategy by summarising existing literature in quality criteria in the three relevant fields: empirical research in social sciences, philosophy/normative ethics research, and EE research. Thus, we composed three subgroups in our Empirical Ethics Working Group (comprising about 13 active members at that time; see endnote (i) for further information about the working group). Each group conducted a review of the methodological literature in the relevant area. We also included explicit recommendations for quality research drafted by scholarly societies (e.g. of psychology or sociology).

Literature was searched with a *narrative*/*selective* search strategy (see e.g. [38]). This strategy was developed due to difficulties and inappropriate results when trying a systematic literature search by using specific search terms, given the interdisciplinary nature of our topic. As a consequence, we decided to broaden our approach by using literature found via PubMed, Philpapers and Google Scholar, via manual search of scientific journals, as well as via expert opinions generated from the members of our working group and their connections to the respective scientific community.

**Figure 2:**
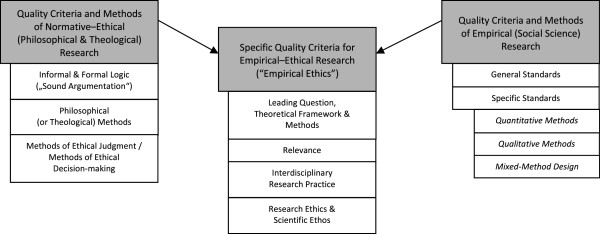
Specific criteria of quality.

The subgroup of trained philosophers (MM, with non-authors JD and UM; see *Acknowledgments*) analysing the criteria within philosophy often had to extract informal and implicitly given criteria (apart from criteria directly related to standards of argument, e.g. logic). The principal subcategories of criteria for philosophical, normative-ethical research are the *criteria of good argument* (as discussed in informal and formal logic (see e.g. [39,40])), the *use of specific philosophical methods* (theories, approaches) with their respective quality criteria (e.g. [41]), and *criteria of good ethical judgement and/or decision-making* (as discussed in models and methods of decision-making (e.g. [42]) (see Figure 2, *Specific criteria of quality*).

The subgroup on quality criteria for empirical research in social sciences (LGR, GR) had to differentiate the literature search of journals and monographs into *general criteria* (such as adequacy of the research process, transparency, good scientific and ethical conduct) and *specific criteria* for *quantitative methods* (e.g. [43]), *qualitative methods* (e.g. [44-48]) or *mixed-methods approaches* (e.g. [49-51]) (see Figure 2).

The subgroup working on quality criteria for EE research (JS, SaS) performed a selective literature review in relevant bioethics journals and books focusing on theoretical and methodological contributions to EE research. While a number of conceptual accounts to EE were identified, the issue of quality standards was only rarely addressed [52,53]. However, parts of the conceptual considerations as identified in the literature could be translated into criteria relevant to EE research [54,55]. In addition, the researchers drew from current EE research addressing ‘end of life issues’ which is performed in an interdisciplinary research group of medical ethicists with a disciplinary background in philosophy, sociology and medicine.

Following these procedures, the findings of each subgroup were discussed within the whole working group. Our next step was to systematise the criteria identified by clustering them into main categories according to their field (empirical, philosophical, EE research), as well as into subcategories (e.g. different categories for qualitative and quantitative empirical research, or different categories for criteria related to logic/argumentation theory and philosophical approaches). The clustering was based on the literature search (inductive strategy), as well as on our own theoretical estimation of aspects relevant for assessing the quality of scientific work (deductive strategy). The summarising process was supported by mind mapping software to track modifications, deletions, additions or re-locations of criteria. The main and sub-categories in this mind map were generated either inductively on the basis of the literature or deductively by own reasoning against the backdrop of scientific experience and theoretical knowledge.

Finally, we derived three overarching standards of scientific research, which were subdivided into formal, cognitive and ethical norms (see below *Three peaks that dominate the scenery*) based especially on the philosophy of science. The actual quality criteria were then seen as specific expressions of these overarching standards.

In step (ii), issues concerning research ethics already found in the reviewed literature were added to the mind map as further criteria. Additional literature was also reviewed [56-59].

A consensus round was initiated for step (iii) where each criterion was again critically discussed with a view to identify possible redundancies. Consensus was reached with the results of the argumentation in the discussion, which was most often accompanied by a final, explicit request if there were any dissenting votes regarding the result. Active members of the working group who were not able to participate at the consensus round (about 3 out of 13 members) had the opportunity to show assent or dissent on the basis of the sent draft of the mind map; there was no crucial dissent that led to a substantial revision of the mind map.

The next step (iv) consisted of focusing on those criteria that were seen as only specific to and coherent with EE research, excluding those relating to broadly empirical research in social sciences and philosophy. With this aim in mind, the working group agreed to divide specific EE criteria into four domains (see also Figure 1), effectively reducing the amount of criteria in the mind map of about 200 (all branches, research ethics included) to about 50. In these four domains, the formal and cognitive norms relevant to all kinds of scientific research were specified and adjusted to the particular field of EE research (“*primary research question, theoretical framework & methods”* and “*relevance”*). Furthermore, the specific interdisciplinary nature of EE research was addressed (*“interdisciplinary research practice”*), and issues of research ethics which are pertinent to EE research (*“research ethics & scientific ethos”*) were considered.

As a result, four new subgroups were established that had to summarise, systematise and elucidate the according criteria on the basis of the already found literature of the three aforementioned subgroups, as well as propose additional criteria if found necessary. The members of these new subgroups also became the authors of the paper at hand and were assigned to the four domains as following: a) *primary research question, theoretical framework & methods* (JI, SiS, SW); b) *relevance* (MM); c) *interdisciplinary research practice* (JS, SaS); and d) *research ethics & scientific ethos* (LGR, GR).

This work also led to the last step (v), the drafting of a refined list which allows the “road map” below to be used as a checklist to guide EE research. For clarity, the criteria included in this list are presented in tabular form. These tables (see below) contain each criterion, operationalised into questions. We decided that it was heuristically more effective to ask questions rather than to consider statements, and to conceive these questions as a pragmatic aid to guide scholars and help them reflect on their own research. Nevertheless, the questions that operationalise criteria should not be understood as simple “yes/no” queries – instead they should function as reflective and critical questions designed to assess certain quality-related aspects of EE research. Each subgroup proposed their phrasing of the questions to the whole author group to achieve consensus on the final phrasing.

This checklist idea is not new. It is already well established in other research fields, e.g. in medicine for guideline recommendations (GRADE [60]; SIGN [61]), quantitative randomised medical trials (CONSORT [62]) and observational epidemiological trials (STROBE [63]), where they are used to check evidence and/or the quality of (the reporting of) trials. Although normative or especially ethical aspects are rarely explicitly mentioned in these checklists, they include implicit normative items such as asking for ethical approval, informed consent, funding or possible sources of bias. Critical appraisal is more and more coming up on the agenda of evidence based medicine ([64] see also [65]).

In analogy, we thought it necessary to render explicit ethical questions that are implicit in EE research. Looking for the best fitting form of presentation, our working group came to the consensus that we would try to adapt the checklist format, as we thought it will be most helpful in order to display the suggested criteria in a clear, feasible way.

## Discussion

### The road map

#### An aerial view: spotting hills and valleys

As is perhaps obvious, the first criteria that have to be considered are *philosophical* quality criteria, and *social sciences* quality criteria. This idea is already mentioned in the EE literature (e.g. [54,66]).

However, even if (ideally) one had knowledge of both sets of criteria, and had the relevant skills to apply them, distinctive features of the quality of EE research would still be missed. This is due to the interdisciplinarity of EE research, and specifically to the complexity that the necessary *methodological combination* of the two sets of criteria requires. This therefore goes far beyond the need for cooperation between disciplines. *Additional* criteria reflect, in particular, the combination of normative-ethical and empirical research.

In the following, the “basic” philosophical and social science quality criteria will not again be summarised, as they have already been discussed in various contexts. We presuppose that quality work in EE research includes consideration of these already established criteria. Instead our major focus will be on criteria that are either (i) specific to EE research (i.e., not directly relevant to other ethical or social sciences research), or (ii) also useful in other research settings, but especially important for EE research.

#### Three peaks that dominate the scenery: “good science” criteria

There are three kinds of standards that dominate all research (including normative-ethical research, empirical research, and combinations of the two), as they are interconnected by norms which are generally concerned with “good” scholarship. They are based on a common-sense definition of what good science is: *formal norms* (scientific writing), *cognitive norms* (general methodology) and *ethical norms* (research ethics). All quality criteria ultimately derive their normative power from the same general norms and can thus be understood (more or less) as specific expressions of these norms.

#### Looking for an interdisciplinary highway

a) Setting up the road signs: designing a primary research question and selecting a theoretical framework and corresponding methods

EE research often faces the problem that the ethical and empirical aspects motivating our research are intertwined [66]. Special attention must therefore be paid to the design and development of the research question. The empirical and normative-ethical aspects of the research question have to be separated clearly without being split into two separate research approaches [22]; at the same time, the relationship between them has to be elucidated [23]. This reflective intermediate step is relevant because it shows why they are part of the *same* research, and cannot be dealt with sufficiently by two (or more) separate research projects. The inherent link between the parts should be considered on several levels: the theoretical assumptions [67], the relevance of empirical knowledge and data to the ethical question and vice versa [15], the chosen methodology [66,68], the type of result/data envisaged, and the way the result can inform further EE research, both empirical and ethical [69] (see Table 1).

**Table 1:** Criteria related to primary research question and selecting a theoretical framework and corresponding methods

**Reflection on the relationship between empirical and ethical–normative/ethical–descriptive research questions** (even if the ethical motivation is more prominent than the ethical research question)	• Can an explicit distinction be made between the empirical and ethical research questions? (e.g. a distinction between interviewing patients about their wishes and the ethical weight given to patient autonomy) [22,23]
	• How dependent is the empirical research question on particular ethical background assumptions? (e.g. justification for the selection of a target group for a questionnaire: why do we think their opinion is ethically relevant?) [70]
	• How is the ethical research question dependent on empirical or socio-theoretical background assumptions? (e.g. ethical considerations of vulnerability of a particular group such as pregnant women: what are the underlying anthropological or psychological considerations? Are there any hidden gender-related stereotypes?) [66]
	• What are the explicit and implicit research interests and motivations of the EE researchers? (e.g. is research with dying patients motivated by curiosity or the moral attempt to empower them? Is the researcher motivated to identify possible conflicts of interest or might the research serve mainly to produce more social acceptance of a technology?) [14,69]
	• What kind of epistemic research interest motivates the researcher to combine ethical and empirical research? (e.g. explaining whether the aim is the evaluation of established ethical practice, or of measures taken to improve ethical practice; or whether the aim is ethical theory-building, norm-construction, or legitimization/critique or a particular practice) [15]
**Development/use of theoretical frameworks:**	• How can a theoretical framework be developed; what are the main limitations of the chosen theoretical framework? (e.g. premises and limitations of a principle of autonomy, when analysing macro-social interactions) [22], see contributions in [67]
	• Were potential ambiguities of central concepts considered within the theoretical framework? (e.g. to which extent is the concept of ‘identity’ used differently in current philosophy and in sociology when wanting to analyse the discourse of identity changes by neuroenhancement empirically and its ethical implications) [71,72]
	• How does the chosen medico-theoretical framework (e.g. concept of disease/health) fit into the ethical-normative framework? (e.g. does a science-positivistic concept of disease fit into a Kantian or hermeneutic approach of ethics?) [73]
	• How does the chosen sociological-, cultural- or philosophical framework (e.g. concept of personal identity) fit into an ethical normative framework (e.g. approach to a cosmopolitical ethics of justice?) [74]
**Use of empirical and ethical methods and their relationship to the theoretical framework:**	• Are the chosen empirical methods compatible with the combined theoretical framework? (e.g. are interviews with doctors as experts compatible with a liberal, autonomy-driven approach that claims to empower patients?)
	• What is the advantage of the chosen method in comparison to other available methods? (e.g. why and when to choose a deductive approach in applied ethics to assess ethical problems of a new technology and not an inductive, or hermeneutic one?) [66,68]
	• Are the chosen methodological approaches appropriate for the envisaged combined research question? (e.g. does the empirical method of interviewing parents generate results relevant for the ethical question of whether parents should be allowed to influence the genetic make-up of their children?)

In theory-guided research, the research questions as well as the chosen methodology normally depend on a particular theoretical framework. The underlying assumptions and theoretical background of both the ethical and the empirical parts of the research should be made explicit and transparent [22,67] (see Table 1). This includes theoretical work on different levels. Firstly, when combining empirical and ethical approaches, we need to reflect on the compatibility of the theories used in each part. The combination of two theoretical approaches needs to be consistent. For example, the empirical discourse analysis of communication structures cannot simply be transformed into ethical questions of individual responsibility for decisions. A more appropriate theoretical ethical approach would be one that ascribes a high level of normative relevance to communication and social interrelations. An analysis of the core concepts of the theoretical approaches makes it possible to test whether these approaches are compatible [73]. These core concepts include ideas such as human agency, the relationship between social and individual levels of agency, concepts of causality, the relevance and concept of gender, to name just a few examples [71,74].

Theoretical compatibility is also a point to consider when it comes to the question of how empirical and ethical research inform each other. Connected to this, the selection of both the empirical methods and the theoretical line of ethical argument (in short, the theoretical method) is crucial. Both methods need to be compatible with the combined theoretical framework of the research, but should also reflect how the results of each part can inform the other part [66,68]. For example – and this is far from being an exhaustive list –, discursive ethical approaches have strong links with argumentative, discursive methods of surveying opinion, while liberal-utilitarian ethical approaches tend to accept opinion polls and quantitative surveys. Critical reflection on the chosen methods and on their limitations for the combined argumentation is a must for all EE.

b) “Driving only when necessary”: demonstrating relevance

Even if an EE study is sufficiently transparent with regard to its primary research question and methods, it can still be *unnecessary*, or more specifically, *not valuable*[22]. From an epistemic, ethical and even economic point of view (due to limited research resources), one can claim that an interdisciplinary approach should only be favoured if it is likely to lead to new insights, or to broader or more nuanced insights than those gained from intradisciplinary research. One should also bear in mind that it is ethically problematic to expose research participants (e.g. patients as interviewees) to stress if the research has low relevance (see also section d).

Relevance should be understood on two levels, as *epistemic relevance* on the one hand and *societal relevance* on the other. Scholars conducting EE research must be able to demonstrate the value of their planned project in at least one sense, and ideally in both (see Table 2).

**Table 2:** Criteria related to relevance

**Contribution to (scholarly) ethics (epistemic/scientific relevance):**	• Will the study possibly produce knowledge that could not be generated by relying on traditional disciplinary methodologies? (e.g. overcoming too separated empirical research and separate philosophical discussion) [15,22]
	• Does the study aim to increase our knowledge, and if so, with regard to what? (e.g. does the study contribute to a balance between theoretically generated norms and empirically found norms? E.g. Does it revise/improve the impact of ethical guidelines?) [56-58,78]
	• Does the study aim to give input on an ongoing controversy, or does it provide a new perspective on it? (e.g. clarifying if relatives are able to give substitute judgment for incapacitated patients or not, or e.g. if post-trial access should be compelling on the basis of new evidence of consequences when post-trial access is not given etc.) [16], partly [76]
	• Does the study aim to offer substantial arguments for or criticism of established ethical positions? (e.g. is a contribution to theory modification or to a refinement of the application of a theory expected? Are descriptive presuppositions of an ethical position, such as anthropological, sociological or psychological assumptions, criticised?) [1,16,66]
	• Does the study aim to contribute to the development or refinement of scientific methods, especially methods of EE research, and if so, how? (e.g. pilot testing of a jointly developed research instrument, identifying the need of developing new or refined forms of interactions between researchers) [6,58]
	• Does the study aim to offer potential for innovation, and if so, what kind of innovation? (e.g. is it a contribution to theory-building expected? Will the study generate new instruments for ethical decision-making?) [6,77]
	• Does the study aim to contribute to another scientific and/or ethical discourse? (e.g. does it contribute to social sciences discourses?) [partly [1]
	• Does the study clearly states to whom it is addressed, and who will benefit from its results? (e.g. are the addresses and/or beneficiaries physicians, nurses, social scientists, ethicists or especially empirical ethicists? Are policy-makers or persons in a management position addressed? [58]
**Contribution to ethical practice (societal/practical relevance):**	• Does the study aim to improve ethical decision-making? (e.g. will it produce evidence that was absent, or will it give guidance regarding the specification of accepted norms or regarding the interpretation of institutional or legal rules?) [6,14,75], partly [17]
	• Does the study aim to raise awareness (among actors, institutions or society) of particular ethical problems? (e.g. does the study identify new ethical problems, or does it highlight specific aspects of a known ethical problem that was not yet addressed sufficiently in practice?) [20,78]
	• Does the study aim to lead to a shift in structures and/or decision-making processes (in relevant institutions)? (e.g. establishing new guidelines or building new forms of committees for ethical review) [77], partly [23]
	• Does the study aim to establish minimum ethical standards (in relevant institutions or professions)? (e.g. creating new informed consent procedures for specific patient groups?)
	• Does the study aim to contribute to or stimulate public debate? (e.g. about physician-assisted suicide, rationing in health care, public health funds etc.)
	• Does the study aim to contribute to or stimulate a process of legislation? (e.g. proposing alteration of legal norms)
	• Does the study aim to articulate the need for reforms (in a certain institution or system of society)? (e.g. by evaluating current practices.) [6]
	• Does the study aim to articulate new ethically pertinent ecological or economic problems? (e.g. costs related to a broad implementation of the use of social robots in elderly care) [16]

The main issue regarding *epistemic relevance* (e.g. [56-58,79]) is whether the study makes a contribution to academic ethics, for example by adding new knowledge (e.g. developing a sound ethical argumentation for the topic in question), contributing to an ongoing controversy (e.g. providing a new perspective), or criticizing established positions on a theoretical or applied level. In other words, it needs to be clear what knowledge gains the research will provide in terms of the development, modification or application of theory.

Another way to ensure scientific relevance, especially in EE research, is to develop or refine scientific methods. Potential for innovation (e.g. constructing a new theory or new instruments for ethical decision-making) should also be regarded as part of epistemic relevance. If there is a contribution to other disciplines (e.g. to social science debates on agency or social dependency), this also fulfils the criterion of epistemic relevance. Finally, the target group of a study, e.g. the future beneficiaries of the results and conclusions of this particular EE research, should be clearly stated. This gives guidance for research planning, and for the analysis of the results.

As far as *societal* or *practical relevance* (e.g. [58,59,79]) is concerned, EE research should be able to show what it contributes to the improvement of ethical praxis. As much ethics research is funded by public money, it might even be argued that there is a moral obligation to generate not only scientifically valuable knowledge, but also knowledge that benefits society or certain groups within it, e.g. specific vulnerable group. This assumption relies on a normative understanding of ethics as a discipline aimed at providing orientation knowledge, which is needed to detect problems or to indirectly or directly improve praxis. So scholars should try to demonstrate the societal/practical relevance of their EE research whenever feasible.

Examples of this kind of relevance could be improvements to ethical decision-making (e.g. an empirically tested model of ethical decision-making), raising awareness of ethical problems and challenges (e.g. showing that without regulated antimicrobial stewardship, there is a high risk of antibiotics increasingly losing their effectiveness), a shift in structures and decision-making processes (e.g. not asking relatives what *they* want, but what *the patient* would have wanted), or the establishment of minimum ethical standards in the institutions or professions related to the praxis under consideration (e.g. formulation and implementation of guidelines). Furthermore, societal relevance is present when the study initiates or simply provides a stimulus for public debate, or leads to/assists the process of legislation. Articulating a need for reforms or new ethically pertinent ecological or economic problems – or articulating existing problems in a new, enlightening way – is also of societal relevance (e.g. [80]).

We are not arguing that all EE research has to demonstrate societal or practical relevance. EE research can be relevant even if it is only relevant for scientific/scholarly reasons. Nonetheless, as stated above, since ethical research seems (to us) to be ultimately guided by a practical interest in ameliorating (some) social practices, scholars need to think about potential societal relevance when planning or evaluating their own EE research (compare also [24]).

c) “Enabling car sharing”: guaranteeing interdisciplinary research practice

Quality criteria for interdisciplinary cooperation in EE research encompass different stages of the research process, including drafting, data gathering, data analysis and conclusions [36,37]. For the different stages, the following points are of particular importance (see Table 3).

**Table 3:** Criteria related to interdisciplinary research practice

**Research drafting:**	• What form of interdisciplinary collaboration serves the needs of an EE study? (e.g. how strong and how often should collaborators interact? Is it necessary to have face-to-face-meetings? Who has to be involved in which step of the research? Is there reflection on the potentials and the limitations of the kind of collaboration?) [18]
	• How can the participating researchers be adequately selected? (e.g. Which disciplines/methods are actually needed?)
	• How can an appropriate task schedule be developed? (e.g. at which point in time is empirical data to be gathered)
	• Which agreements must be reached with regard to interdisciplinary communication? (e.g. consideration of terms used, explanation of professional jargon and development of a “common language”) [3]
	• How can competencies and responsibilities be reasonably distributed within the research team? (e.g. despite their varying competencies, will all the interdisciplinary researchers remain actively involved in the research process? Who is accountable for what?)
	• How can research questions be developed jointly? (e.g. regarding different interests and disciplinary perspectives, or regarding the goal of the study)
	• How can the literature search be carried out? (e.g. having to acknowledge empirical-ethical studies from one’s own thematic field as well as to acknowledge both empirical and ethical work from different disciplines in diverse types of publication)
**Data gathering:**	• How is the joint development or modification of a research instrument carried out? (e.g. Is there a process that allows for dissent and argument in developing or modifying a research instrument?)
	• Is there normative-ethical reflection on the empirical research process? (e.g. can implicit normativity be revealed that is related to a theoretical background (“social constructivism”)?)
	• Is there a mutually critical appraisal by normative and empirical sciences with regard to data gathering? (e.g. what constitutes “good” data for the EE study) [54]
**Data analysis and conclusions:**	• How do normative and empirical aspects interrelate with regard to analysis and deliberation?
	◦ Is the analysis of the empirical data influenced by normative theories, concepts, or standpoints? (e.g. by a specific account of patient autonomy)
	• Is the normative deliberation influenced by the requirements of the empirical data analysis? (e.g. by standardization of data) [81]
	• How do normative and empirical aspects interrelate with regard to the study’s conclusions?
	◦ Are the ethical conclusions actually linked with normative premises? (e.g. avoiding an is/ought fallacy) [16,23]
	◦ Are the empirical conclusions supported by the data, or is there a bias in the empirical results based on the normative conclusions? (e.g. avoiding a normativist fallacy or “wishful thinking”, deducing broad conclusions from fine-grained data, under- or overrating of empirical data, ignoring of empirical evidence that would criticize normative conclusions etc.) [82,83]
	• Is there a critical evaluation of the results? (e.g. addressing methodological critique with regard to interdisciplinary cooperation, or indication of limitations)

In the *planning stage* of an EE study, the reflection on adequate forms of interdisciplinary collaboration should include consideration of not only its potential benefits, but also its limitations. Limitations are often the result of different professional jargons and terms [3]. It is therefore advisable to clarify terminology at the beginning of the collaboration. Furthermore, a clear distribution of competences and responsibilities within the research team is needed. A further challenge in the planning of an EE study is posed by the literature research, which has to consider journals, books, and databases from diverse fields [84]. During *data gathering*, it is important to reflect the potential bias produced by normative or descriptive assumptions [54]*.* One should also be aware of possible biases when selecting published empirical studies (e.g. [85]).

During *data analysis*, researchers should explicitly discuss the relevance of ethical theories, concepts, or standpoints for the empirical data analysis, as well as possible reductionist tendencies associated with the requirements of particular methods, e.g. the standardization of question wording and statistical analysis in quantitative surveys [81]. A final crucial question is how much the empirical results contribute to the normative judgement [16,24]. Do they help to “justify” a particular norm, and if so, based on which ethical theoretical consideration? Or do they have an impact on the level of practical application, when public acceptance seems crucial if a rule is to be applied in a particular way? It is also desirable to consider whether the interpretation of the empirical data (e.g. approval or criticism of a particular group’s view) was led by a particular ethical view [82].

d) “Driving responsibly”: observing research ethics & scientific ethos

Of course EE research is liable to general principles of research ethics in general and bioethics in particular. Important topics are avoiding harm to the participants [86,87], confidentiality and data safety [86,88,89], informed consent [86,90,91], management of research data [89,92].

Our discussions within our broader research group revealed, that in addition to that some aspects are of special importance in the field of EE research:

In an interdisciplinary setting where researchers can have different institutional backgrounds and differ in the personal motivations for their research, potentially competing interests should be disclosed and critically discussed. There should be explicit reflection about the interdisciplinary setting and the division of labour (see e.g. [93]). This, we think, must include a readiness to critically reflect upon a possible *inclination* to design the empirical part of the research in such a way that the results may favour the researcher’s own ethical position. For example, one should ask: am I especially critical or joyful when it comes to this issue? Do I tend to exaggerate the issue in my research questions? Which results of the empirical research would I predict and which would I wish to see? Here the interdisciplinary context offers an excellent opportunity for an open discourse (see Table 4).

**Table 4:** Criteria related to research ethics & scientific ethos

**Competing interests:**	• Which personal (e.g. cultural, philosophical, theological) presumptions concerning ethics may bias the EE research process significantly and how can they be adequately managed? (e.g. inclination to a emotivist meta-ethics, a neopositivist philosophy of science, a postmodernist account of society etc.) [42]
**Informed consent:**	• Do different standards exist in the various disciplines involved, and if so, have they been critically and respectfully discussed among the EE research team to find the most appropriate ethical standard? (e.g. is waiving of consent allowed, is assent sufficient, how to establish informed consent in emotional difficult situations at the end of life etc.) [43-49]
	• Is the EE research team aware of a possible confidence bonus, and have strategies been developed to deal with this phenomenon carefully? (e.g. making transparent which goals and which limitations the own study will have and informing research participants and partners accordingly)
**Reporting results:**	• Is the EE research team aware of the (implicit) ethical impact of the way results are presented? (e.g. was the potential for stigmatization or discrimination considered when choosing the wording and emphasis of particular results?) [30], partly [50]
	• Has the EE research team made sure that the way the results are presented reduces the potential for misinterpretation by third parties such as politicians and special interest groups? (e.g. by changing perspectives when re-reading results and revising the wording etc.)
**Consequences for the future:**	• Has the risk–benefit ratio for the EE research project been discussed in terms of its possible consequences for the people/society of the (near/more distant) future? (e.g. does lay considerable burden on study participants for a relatively low practical or epistemic output?) [30]
	• Has the research team overlooked any negative consequences that could be detected in advance and therefore avoided? (e.g. acknowledging non-scientific partners when publishing, the handling of emotional distress of participants in interview studies with sensible questions, supervision of researchers involved in asking sensible questions etc.)

With regard to so-called *informed consent*, different standards may obtain in different disciplines such as medicine [94,95], psychology [96,97] or social sciences [98-100]. However, we think that it is the task of the interdisciplinary research team to openly and ethically consider the possibilities and to choose the most appropriate format – which may exceed the legal standard. Regardless of consent by the participants, the definitive ethical responsibility remains with the researcher – especially as the EE researcher may be faced with a *confidence bonus* granted by the research participants just because she/he is an ethicist (an “ethical misconception” analogous to the “therapeutic misconception” in some clinical research). It is the researcher’s duty to deal with this bonus very carefully.

EE research can be misinterpreted by politicians and special interest groups. Therefore, we suggest that researchers should reflect on the following questions: have we ensured that the results cannot easily be misunderstood or misused? Might the EE study have unanticipated negative consequences that could be detected in advance and therefore avoided? Although it seems clear that we cannot anticipate every kind of negative consequences, the EE researcher may have a particular responsibility to carefully reflect on the outcomes of her/his own research beyond the short time frame of the study, since EE might have a strong influence on public policies, e.g. in health care or biopolitics.

### Limitations and conclusions

#### Limitations

We assume that it is theoretically acceptable to start from the analytical premise that there is genuine ethical research on one side and genuine empirical–descriptive research on the other, and that these have to be paired up with each other through interdisciplinary research practices. We reject, on the other hand, a stance that denies the need for, the possibility of, or the value of strong interdisciplinary collaboration between empirical and ethical research (e.g. being afraid of naturalistic fallacies) to gain a valid *applied* ethical conclusion^f^.

Our research is strongly influenced by experience and in-depth analysis of current EE research in the context of Western bioethics and medical ethics. It does not encompass other possible epistemic approaches such as revealing “concealed” normativity in empirical research, or more institutional aspects of a combination of ethical and empirical disciplines. Our “road map” is therefore restricted to this field. Whether this “road map” can be of use in other ethical disciplines, such as economic/business ethics or ecological ethics, has to be explored elsewhere.

Because our literature search strategy was selective, there are of course limitations concerning the completeness of reviewed literature. There is a good case to believe that further criteria could be mentioned when including additional literature. But discussing quality criteria for EE research in a single short publication necessitates condensation and simplification; we therefore understand this paper as an attempt to encourage more intensive meta-ethical and methodological discussion within the EE field.

The proposed criteria in this paper need to be tested in EE research practice. It is likely that the deliberate use of these criteria to guide and report on individual research will lead to the refinement, addition or removal of some criteria.

## Conclusions

EE is a highly interdisciplinary and dynamic research field with specific methodological aspects. Because of its genuinely interdisciplinary nature, a reflection on methodological quality is necessarily more complex than in traditional intradisciplinary ethical or empirical research. However, contributions addressing the specific challenges of EE research are so far rare. We have therefore tried, in this article, to give an overview of basic criteria which have to be considered to arrive at EE studies of good scientific quality; they may also inform the assessment of research protocols or proposals. We assume that many of the suggested criteria are self-evident, perhaps even common-sense – but the important thing here is the argument that they should be used *together as a whole*.

However, our analysis also points out conflicts that may occur between different quality criteria, especially those concerning the empirical part of the research on the one hand and the ethical part on the other. While researchers may not always be able to overcome such conflicts, it is paramount to at least address these different requirements when publishing EE research and to defend the chosen approach in the light of this conflict.

## Summary

We have argued that empirical research in EE is not an end in itself, but vital for reaching applied normative conclusions. As such EE research is usually interdisciplinary, engaging in sound EE research requires more than merely maintaining the quality of normative argument and empirical method.

Thus criteria for determining the quality of genuinely interdisciplinary aspects of EE research, methodological as well as ethical, are required. We have proposed several criteria of this kind under the headings *Primary Research Question, Theoretical Framework and Methods*, *Relevance*, *Interdisciplinary Research Practice* and *Research Ethics and Scientific Ethos*. Although these criteria cannot (yet) be considered definitive, they provide a starting point for reflection on the topic. The criteria need to be tested in real EE research practice and evaluation, and are likely to require further refinement.

## Endnotes

^a^The following paper has been jointly produced by core members of the *Empirical Ethics Working Group* (coordinator until the end of 2013: Prof. Dr Silke Schicktanz; current coordinators: Jan Schildmann and Marcel Mertz) of the German Academy for Ethics in Medicine (AEM; see http://www.aem-online.de). The working group was founded in 2007, with the objective of bringing together researchers interested in the challenges of empirical research in bioethics, and particularly its relevance to and connection with normative-ethical research. The working group became highly interdisciplinary, consisting of philosophers, theologians, medical ethicists, social scientists, physicians, and humanities scholars.

^b^Researchers in the field of EE use a broad variety of empirical research approaches, which overlap with the methods used in empirical disciplines such as the social sciences, psychology, ethnography and anthropology (see e.g. [16,101]).

^c^Though sometimes results from empirical research in bioethics, without any normative conclusions, are also categorised under this label (see e.g. [55,102,103]).

^d^This is especially important for attempts to ethically improve clinical practice guidelines, ethical guidelines or clinical support services by relying on empirical data, as the situation concerning quality criteria is similarly unsatisfactory (e.g. [77,104]).

^e^Studies that explicitly rely on (vast) empirical research (results) for their ethical argumentation, but do not engage in empirical research themselves, should also be considered when discussing issues of quality in EE research. We decided, though, to focus here solely on EE studies that incorporate genuine empirical research.

^f^Though we do not think that EE research is generally burdened with the problem of avoiding the naturalistic fallacy, or that it usually illegitimately crosses the is/ought gap (see [23]).

## Abbreviations

EE: Empirical ethics.

## Competing interests

*Financial competing interest:* None to declare. *Non-financial competing interest:* All the authors of this paper are committed to EE research and will therefore not *generally* assess such efforts in a negative way. Moreover, they have all engaged in EE research and have an interest in pursuing such research further.

## Authors’ contributions

MM: drafted and revised the manuscript in general, drafted section “Driving only when necessary”, coordinated the writing process, and finalised the manuscript. JI: drafted section “Setting up the road signs”, and revised the manuscript. GR: drafted section “Driving responsibly”, made substantial contributions to limitations section, and revised the manuscript. LGR: drafted section “Driving responsibly”, made substantial contributions to limitations section, and revised the manuscript. SaS: drafted section “Enabling car sharing”, made substantial contributions to conclusions section, and revised the manuscript. JS: drafted section “Enabling car sharing”, made substantial contributions to conclusions section, and revised the manuscript. SW: drafted section “Setting up the road signs”, and revised the manuscript. SiS: drafted section “Setting up the road signs”, revised and finalised the manuscript; initiated and coordinated the Empirical Ethics Working Group (until the end of 2013). All the authors were involved in the conception and design of the material presented, and read and approved the final manuscript.

## Pre-publication history

The pre-publication history for this paper can be accessed here:

http://www.biomedcentral.com/1472-6939/15/17/prepub
